# Effect of the 3'APOB-VNTR polymorphism on the lipid profiles in the Guangxi Hei Yi Zhuang and Han populations

**DOI:** 10.1186/1471-2350-8-45

**Published:** 2007-07-17

**Authors:** Yin Ruixing, Chen Guangqin, Wang Yong, Lin Weixiong, Yang Dezhai, Pan Shangling

**Affiliations:** 1 Department of Cardiology, Institute of Cardiovascular Diseases, the First Affiliated Hospital, Guangxi Medical University, Nanning, China; 2 Department of Molecular Biology, Guangxi Medical Scientific Research Center, Nanning, China; 3 Department of Pathophysiology, School of Premedical Sciences, Guangxi Medical University, Nanning, China

## Abstract

**Background:**

Apolipoprotein (Apo) B is the major component of low-density lipoprotein (LDL), very low-density lipoprotein (VLDL) and chylomicrons. Many genetic polymorphisms of the Apo B have been described, associated with variation of lipid levels. However, very few studies have evaluated the effect of the variable number of tandem repeats region 3' of the Apo B gene (3'APOB-VNTR) polymorphism on the lipid profiles in the special minority subgroups in China. Thus, the present study was undertaken to study the effect of the 3'APOB-VNTR polymorphism on the serum lipid levels in the Guangxi Hei Yi Zhuang and Han populations.

**Methods:**

A total of 548 people of Hei Yi Zhuang were surveyed by a stratified randomized cluster sampling. The epidemiological survey was performed using internationally standardized methods. Serum lipid and apolipoprotein levels were measured. The 3'APOB-VNTR alleles were determined by polymerase chain reaction (PCR) followed by electrophoresis in polyacrylamide gels, and classified according to the number of repeats of a 15-bp hypervariable elements (HVE). The sequence of the most common allele was determined using the PCR and direct sequencing. The possible association between alleles of the 3'APOB-VNTR and lipid variables was examined. The results were compared with those in 496 people of Han who also live in that district.

**Results:**

Nineteen alleles ranging from 24 to 64 repeats were detected in both Hei Yi Zhuang and Han. HVE56 and HVE58 were not be detected in Hei Yi Zhuang whereas HVE48 and HVE62 were totally absent in Han. The frequencies of HVE26, HVE30, HVE46, heterozygote, and short alleles (< 38 repeats) were higher in Hei Yi Zhuang than in Han. But the frequencies of HVE34, HVE38, HVE40, homozygote, and long alleles (≥ 38 repeats) were lower in Hei Yi Zhuang than in Han (*P *< 0.05–0.01). The levels of total cholesterol (TC), high-density lipoprotein cholesterol (HDL-C) and Apo B in Hei Yi Zhuang but not in Han were higher in VNTR-LS (carrier of one long and one short alleles) than in VNTR-LL (the individual carrying two long alleles) genotypes. The levels of TC, triglycerides (TG), LDL cholesterol, and Apo B in Hei Yi Zhuang were higher in both HVE34 and HVE36 alleles than in HVE32 allele. The levels of TC, TG, HDL-C and Apo B in Hei Yi Zhuang were also higher in homozygotes than in heterozygotes. There were no significant differences in the detected lipid parameters between the VNTR-SS (carrier of two short alleles) and VNTR-LS or VNTR-LL genotypes in both ethnic groups.

**Conclusion:**

There were significant differences of the 3'APOB-VNTR polymorphism between the Hei Yi Zhuang and Han populations. An association between the 3'APOB-VNTR polymorphism and serum lipid levels was observed in the Hei Yi Zhuang but not in the Han populations.

## Background

Coronary heart disease (CHD) is the main cause of death in most industrialized nations, and is of growing concern in developing countries. Disorders of lipid metabolism such as elevated serum levels of total cholesterol (TC) [1-3], triglycerides (TG) [4,5], low-density lipoprotein cholesterol (LDL-C) [6,7], and apolipoprotein (Apo) B [8-11] have been considered important risk factors in the pathogenesis of atherosclerosis and CHD [12]. Apo B is the main protein component of LDL, very low-density lipoprotein (VLDL) and chylomicrons and plays an important role in cholesterol metabolism [13,14]. It is involved in the assembly and secretion of chylomicrons from the small intestine and VLDL-C from the liver [15]. The Apo B gene is a tissue-specific gene that is expressed mainly in liver and intestine [16,17]. Numerous polymorphisms of the Apo B gene have been described. Particularly, a variable number of tandem repeats (VNTR) polymorphism, which is located 75 bp downstream the second polyadenylation signal at the 3' end of the Apo B gene (2p24-p23) has been found to be common in some ethnic groups [18-21], and it has been reported to be associated with modifications of lipid concentrations [22-28] and the risk of CHD [25-30] in some studies but not in others [31,32].

There are fifty-six nationalities in China. Han is the largest nationality, and Zhuang is the largest minority. Geographically and linguistically, Zhuang can be classified into 43 ethnic subgroups, among which Hei Yi (means black-worship and black dressing) Zhuang is proved to be the most conservative subgroup. They hold that black color is beautiful, and they like to wear black garments and pants. Black color has become the marking of Hei Yi Zhuang. The population of Hei Yi Zhuang is 51,655. Because of remoteness, continuous mountains, blocking, abominable environment, as well as strict intra-ethnic marriages, the special customs and culture are still preserved up to now. We have previously reported the differences of the serum lipid parameters [33,34], and the effects of the microsomal triglyceride transfer protein gene and the lipoprotein lipase gene polymorphism at *Pvu *II locus on the lipid profiles between the Hei Yi Zhuang and Han populations [35,36]. The aim of the present study was to assess the effect of the 3'APOB-VNTR polymorphism on serum lipid levels in the Guangxi Hei Yi Zhuang and Han populations.

## Methods

### Subjects

A total of 548 people of Hei Yi Zhuang who reside in 7 villages in Napo County, Guangxi Zhuang Autonomous Region were surveyed by a stratified randomized cluster sampling. The age of the subjects ranged from 15 to 80 years, with an average age of 45.78 ± 16.33 years. There were 290 males (52.92%) and 258 females (47.08%). All subjects were peasants. At the same time, a total of 496 people of Han who reside in 9 villages in Napo County were also surveyed by the same method. The average age of the subjects was 45.24 ± 15.87 years (range 15 to 82). There were 264 males (53.23%) and 232 females (46.77%). All of them were also peasants. All of these subjects were essentially healthy and had no evidence of diseases related to atherosclerosis. None of them had been treated with β-adrenergic blocking agents and lipid-lowering drugs such as statins or fibrates. The present study was approved by the Ethics Committee of the First Affiliated Hospital, Guangxi Medical University. Informed consent was obtained from all subjects after they received a full explanation of the study.

### Epidemiological survey

The survey was carried out using internationally standardized methods, following a common protocol. Information on demographics (age, gender, and residential area), socioeconomic status (education level achieved, marital status, and annual household income), cigarette smoking, alcohol consumption, and physical activity was collected with a standardized questionnaire. Smoking status was categorized into groups of cigarettes per day: <20 and ≥ 20. Alcohol consumption was categorized into groups of grams of wine per day: ≤ 250 g and >250 g. The physical examination included several anthropometric parameters such as blood pressure, body height, body weight, and waist circumference etc., and body mass index (BMI) was calculated as weight (kg) divided by height (m) squared. Sitting blood pressure was measured three times with the use of a mercury sphygmomanometer after the subject rest 5 minutes, and the average of the three measurements was used for the level of blood pressure. Systolic blood pressure was determined by the first Korotkoff sound, and diastolic blood pressure by the fifth Korotkoff sound.

### Measurements of lipids and apolipoproteins

A venous blood sample of 8 mL was obtained from all subjects between 8 and 11 AM, after at least 12 hours of fasting, from a forearm vein after venous occlusion for few seconds in a sitting position. 3 mL was collected into glass tubes and allowed to clot at room temperature, and used to determine serum lipids, and the remaining 5 mL was transferred to tubes with anticoagulate solution (4.80 g/L citric acid, 14.70 g/L glucose, and 13.20 g/L tri-sodium citrate) and used to extract DNA. Immediately following clotting serum was separated by centrifugation for 15 minutes at 3000 rpm. The levels of TC, TG, high-density lipoprotein cholesterol (HDL-C), and LDL-C in samples were determined by enzymatic methods with commercially available kits, Tcho-1, TG-LH (RANDOX Laboratories Ltd., Ardmore, Diamond Road, Crumlin Co. Antrim, United Kingdom, BT29 4QY), Cholestest N HDL, and Cholestest LDL (Daiichi Pure Chemicals Co., Ltd., Tokyo, Japan), respectively. Serum Apo A1 and Apo B levels were assessed by the immunoturbidimetric immunoassay using a commercial kit (RANDOX Laboratories Ltd.). All determinations were performed with an autoanalyzer (Type 7170A; Hitachi Ltd., Tokyo, Japan) in the Clinical Science Experiment Center of the First Affiliated Hospital, Guangxi Medical University.

### Deoxyribonucleic acid extraction

Genomic deoxyribonucleic acid (DNA) was extracted from the peripheral blood leukocytes by the phenol-chloroform method as our previous reports [35,36]. The extracted DNA was stored at 4°C until analysis.

### Determination of the 3'APOB-VNTR polymorphism

#### Amplification of genomic DNA

Apo B 3'VNTR amplification was carried out by polymerase chain reaction (PCR) using a forward and reverse oligonucleotide primer encompassing the entire Apo B 3'VNTR sequence according to the previously reported protocol [29,37-39]. The sequence of the forward and backward primers used was 5'-ATGGAAACGGAGAAATTATG-3' and 5'-CCTTCTCACTTGGCAAATAC-3'. Each amplification reaction was performed with 100 ng (3 μL) of genomic DNA; 1.0 μL of each primer (20 pmo1); 5 μL of 10 × buffer solution; 4 μL dNTP; and 1.0 μL (2 U) Taq polymerase a total volume of 50 μL. For the amplification, initial denaturation at 94°C for 5 minutes was followed by 30 cycles of denaturation at 94°C for 40 s, annealing at 58°C for 80 s, and extension at 72°C for 1 minute, with final extension at 72°C for 10 min. The amplified products were directly observed after electrophoresis in 2% sepharose gel.

#### Genotyping of the 3'APOB-VNTR polymorphism

To separate the 3' VNTR alleles, 15 μL of amplified DNA were run on 6% polyacrylamide gel at 20 mA for 5 hours. Stained with ethidium bromide, the gel was visualized under UV light and photographed. The length of each amplified DNA fragment was determined by comparing migration of a sample with that of standard DNA marker. The number of tandem repeats was calculated using the equation: repeat number = [fragment length (bp) - 138 bp] / 15 bp. The nomenclature proposed by Ludwig et al [39] was used in this study. One short DNA sequence at the 3' boundary of the hypervariable region was regarded as a repeat [40].

#### DNA sequencing

Genomic DNA was chosen from the subjects carrying HVE32 of Hei Yi Zhuang and was amplified by symmetric PCR. The PCR product was purified by low melting point gel electrophoresis and phenol extraction. The products were analyzed by using an ABI Prism 3100 (Applied Biosyatems) in our Medical Scientific Research Center, Guangxi Medical University, China.

### Diagnostic criteria

The normal values of serum TC, TG, HDL-C, LDL-C, Apo A1, and Apo B in our Clinical Science Experiment Center were 3.10–5.17, 0.56–1.70, 0.91–1.81, 1.70–3.20 mmol/L, 1.00–1.76, and 0.63–1.14 g/L; respectively. The individuals of TC >5.17 mmol/L and/or TG >1.70 mmol/L should be defined as hyperlipidemic [33,34]. Hypertension was diagnosed according to the criteria of 1999 World Health Organization-International Society of Hypertension Guidelines for the management of hypertension [41]. Uncontrolled hypertension was defined as a systolic blood pressure of 140 mmHg or greater and a diastolic blood pressure of 90 mmHg or greater. The subjects with only systolic blood pressure = 140 mmHg but diastolic blood pressure <90 mmHg were diagnosed as isolated systolic hypertension. The diagnostic criteria of overweight and obesity were according to the Coorperative Meta-analysis Group of China Obesity Task Force. Normal weight, overweight and obesity were defined as a BMI <24, 24–28, and >28 kg/m^2^, respectively [42].

### Statistical analysis

Epidemiological data were recorded on a pre-designed form and managed with Excel software. Levels of the quantitative variables are presented as mean ± standard deviation (SD). The difference of general characteristics between Hei Yi Zhuang and Han was tested by the Student's unpaired *t *test. The allele frequencies of DNA polymorphism of the Apo B gene were estimated by gene counting. A chi-square analysis was used to evaluate the allelic and genotypic frequencies that were calculated from the observed genotypic counts and to assess Hardy-Weinberg expectations. One-way analysis of variance (ANOVA) was used to assess the differences of lipid variables among 3 genotypes. Significant difference was then subjected to multiple comparison using the Newman-Keuls. In order to evaluate the association of serum lipid levels with sex (male = 0; female = 1), age (year), cigarette smoking (nonsmokers = 0; <20 cigarettes/day = 1; ≥ 20 cigarettes/day = 2), alcohol consumption (nondrinkers = 0; ≤ 250 g wine/day = 1; >250 g/day = 2), BMI (kg/m^2^), pulse pressure (mmHg), unconditional logistic regression analysis with forward stepwise modeling was also performed in the population. All statistical analyses were done with the statistical software package SPSS 10.0 (SPSS Inc., Chicago, Illinois). A *P *value of less than 0.05 was considered significant.

## Results

### General characteristics

Table 1 gives the general characteristics of the subjects between Hei Yi Zhuang and Han. The levels of systolic blood pressure and pulse pressure were significantly higher in Hei Yi Zhuang than in Han (*P *< 0.01 for each), whereas body weight and BMI were higher in Han than in Hei Yi Zhuang (*P *<0.05 and 0.01; respectively). There were no significant differences of body height, diastolic blood pressure levels, age and sex constituent ratio between the Hei Yi Zhuang and Han subjects.

**Table 1:** Comparison of general characteristics between the Hei Yi Zhuang and Han populations

Parameters	Hei Yi Zhuang	Han Nationality
Number	548	496
Sex (male/female)	290/258	264/232
Age (year)	45.78 ± 16.33	45.24 ± 15.87
Body height (cm)	152.76 ± 9.57	152.88 ± 8.74
Body weight (kg)	49.13 ± 8.43	50.58 ± 8.62*
Body mass index (kg/m^2^)	21.26 ± 2.12	22.58 ± 2.57**
Systolic blood pressure (mmHg)	125.79 ± 17.36	118.75 ± 16.73**
Diastolic blood pressure (mmHg)	76.86 ± 11.24	76.46 ± 10.36
Pulse pressure (mmHg)	49.14 ± 13.57	44.27 ± 11.62**
Cigarette smoking [n(%)]		
Nonsmoker	352(64.23)	356(71.77)*
<20 cigarettes/day	114(20.81)	66(13.31)*
≥20 cigarettes/day	82(14.96)	74(14.92)
Alcohol consumption [n(%)]		
Nondrinker	246(44.89)	260(52.42)**
≤250 g wine/day	224(40.88)	180(36.29)
>250 g wine/day	78(14.23)	56(11.29)

*P < 0.01 and **P < 0.001 in comparison with Hei Yi Zhuang

### Serum lipid levels

As shown in Table 2, the levels of TC, TG, LDL-C, and Apo B in Hei Yi Zhuang were significantly lower than those in Han (*P *< 0.05–0.01), but the levels of HDL-C in Hei Yi Zhuang were significantly higher than those in Han (*P *< 0.01). There were no significant differences of Apo A1 levels between the two ethnic groups (*P *> 0.05).

**Table 2:** Comparison of serum lipid levels between the Hei Yi Zhuang and Han populations

Nationality/sex	*n*	TC (mmol/L)	TG (mmol/L)	HDL-C (mmol/L)	LDL-C (mmol/L)	Apo A1 (g/L)	Apo B (g/L)
Hei Yi Zhuang	548	4.44 ± 0.96	1.07 ± 0.61	2.53 ± 0.72	2.11 ± 0.55	1.43 ± 0.14	0.86 ± 0.19
Male	290	4.46 ± 0.94	1.14 ± 0.75	2.63 ± 0.74	2.09 ± 0.59	1.43 ± 0.14	0.88 ± 0.21
Female	258	4.45 ± 0.96	0.97 ± 0.42^††^	2.48 ± 0.67^†^	2.12 ± 0.51	1.42 ± 0.15	0.85 ± 0.20
Han nationality	496	4.73 ± 1.02**	1.21 ± 0.71**	2.31 ± 0.75**	2.42 ± 0.57**	1.42 ± 0.13	1.19 ± 0.22**
Male	264	4.82 ± 1.16**	1.24 ± 0.83	2.34 ± 0.82**	2.56 ± 0.45**	1.41 ± 0.15	1.32 ± 0.23
Female	232	4.63 ± 0.92^†^*	1.18 ± 0.65**	2.27 ± 0.67**	2.36 ± 0.42^††^**	1.43 ± 0.12	0.98 ± 0.21^††^**

TC, total cholesterol; TG, triglycerides; HDL-C, high-density lipoprotein cholesterol; LDL-C, low-density lipoprotein cholesterol; Apo A1, apolipoprotein A1; Apo B, apolipoprotein B; ^†^*P *< 0.05 and ^††^*P *< 0.01 in comparison with male of same nationality; *P < 0.05 and **P < 0.01 in comparison with Hei Yi Zhuang

### Allelic frequencies

**Figure 1:**
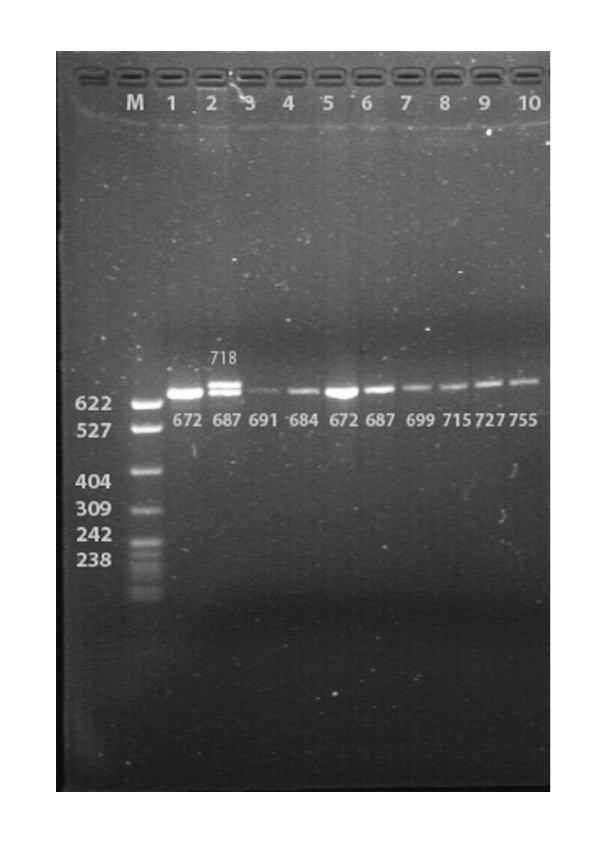
Electrophoretic result of PCR products of the samples in Han. Lane M, 322 bp marker ladder (PBR322/MspI); Lane 2, heterozygote (two straps); Lanes 1, 3–10, homozygotes (one strap)

**Figure 2:**
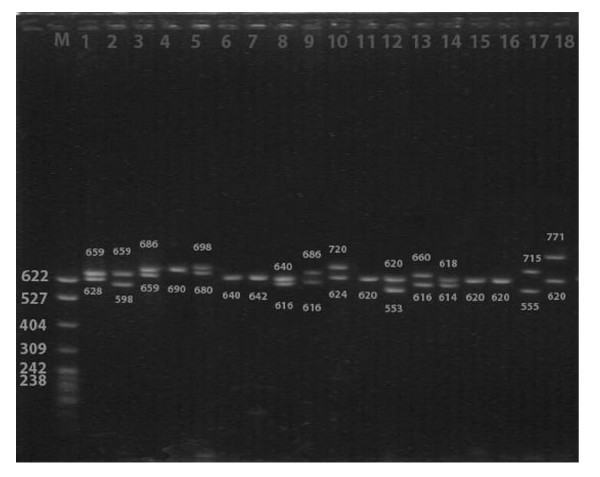
Electrophoretic result of PCR products of the samples in Hei Yi Zhuang. Lane M, 322 bp marker ladder (PBR322/MspI); Lanes 1–3, 5, 8–10, 12–14, 17, and 18, heterozygotes (two straps); Lanes 4, 6, 7, 11, 15, and 16, homozygotes (one strap)

**Figure 3:**
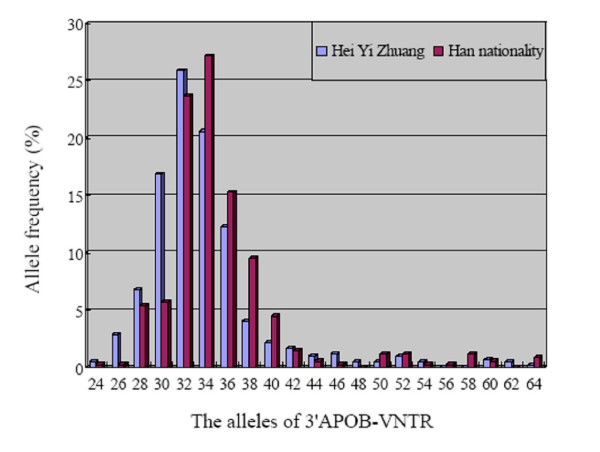
The frequency distribution of the 3'APOB-VNTR alleles between the Hei Yi Zhuang and Han populations; *P *< 0.05 (HVE40 and HVE46) and *P *< 0.01 (HVE26, HVE30, HVE34 and HVE38), in comparison between Hei Yi Zhuang and Han.

Using the PCR followed by electrophoresis in polyacrylamide gels and classifying according to the number of repeats of a 15-bp hypervariable elements (HVE), 19 different alleles of the Apo B gene 3'VNTR comprising from 24 to 64 HVEs were identified in both Hei Yi Zhuang and Han (Table 3, Figures 1 and 2). They were 24, 26, 28, 30, 32, 34, 36, 38, 40, 42, 44, 46, 48, 50, 52, 54, 60, 62 and 64 repeats in Hei Yi Zhuang, and 24, 26, 28, 30, 32, 34, 36, 38, 40, 42, 44, 46, 50, 52, 54, 56, 58, 60 and 64 repeats in Han. HVE56 and HVE58 were not be detected in Hei Yi Zhuang and HVE48 and HVE62 were not be found in Han. The most frequent allele was HVE32 (25.9%), followed by HVE34 (20.6%) and HVE30 (16.9%) in Hei Yi Zhuang, whereas the most frequent allele was HVE34 (27.2%), followed by HVE32 (23.7%) and HVE36 (15.3%) in Han. The frequencies of HVE26, HVE30, HVE46, heterozygote, and short alleles (<38 repeats) were higher in Hei Yi Zhuang than in Han (2.9% vs. 0.3%, *P *< 0.01; 16.9% vs. 5.7%, *P *< 0.01; 1.2% vs. 0.3%, *P *< 0.05; 50.4% vs. 34.6%, *P *< 0.01 and 86.0% vs. 77.8%, *P *< 0.01; respectively), whereas the frequencies of HVE34, HVE38, HVE40, homozygote, and long alleles (≥ 38 repeats) were lower in Hei Yi Zhuang than in Han (20.6% vs. 27.2%, *P *< 0.05; 4.1% vs. 9.6%, *P *< 0.01; 2.2% vs. 4.5%, *P *< 0.01; 49.6% vs. 65.4%, *P *< 0.01 and 14.0% vs. 22.2%, *P *< 0.01; respectively). No allele less than 24 was found in both ethnic groups (Figure 3).

**Table 3:** Comparison of the allelic frequencies of the 3'APOB-VNTR polymorphism in the Hei Yi Zhuang and Han populations [n(%)]

Alleles	Hei Yi Zhuang	Han Nationality
HVE24	4(0.5)	2(0.3)
HVE26	24(2.9)	2(0.3)**
HVE28	56(6.8)	36(5.4)
HVE30	140(16.9)	38(5.7)**
HVE32	214(25.9)	158(23.7)
HVE34	170(20.6)	182(27.2)**
HVE36	102(12.3)	102(15.3)
HVE38	34(4.1)	64(9.6)**
HVE40	18(2.2)	30(4.5)*
HVE42	14(1.7)	10(1.5)
HVE44	8(1.0)	4(0.6)
HVE46	10(1.2)	2(0.3)*
HVE48	4(0.5)	0(0.0)
HVE50	4(0.5)	8(1.2)
HVE52	8(1.0)	8(1.2)
HVE54	4(0.5)	2(0.3)
HVE56	0(0.0)	2(0.3)
HVE58	0(0.0)	8(1.2)
HVE60	6(0.7)	4(0.6)
HVE62	4(0.5)	0(0.0)
HVE64	2(0.2)	6(0.9)
Sum	826(100)	668(100)
≥38	116(14.0)	148(22.2)**
<38	710(86.0)	520(77.8)**

HVE, hypervariable element; *P < 0.05 and **P < 0.01 in comparison with Hei Yi Zhuang

### 3'APOB-VNTR polymorphism and serum lipid levels

By re-coding the 3'APOB-VNTR alleles into long and short alleles, we found that there were significant differences of the long or short allele frequency between Hei Yi Zhuang and Han (*P *< 0.01). The levels of TC, HDL-C and Apo B in Hei Yi Zhuang but not in Han were higher in VNTR-LS (carrier of one long and one short alleles) genotype than in VNTR-LL (the individual carrying two long alleles) genotype. However, there were no significant differences of TC, TG, HDL-C, LDL-C, and Apo Al levels between VNTR-SS (carrier of two short alleles) genotype and VNTR-LS or VNTR-LL genotype in Hei Yi Zhuang. There were also no significant differences of TC, TG, HDL-C, LDL-C, Apo Al and Apo B levels among the three genotypes in Han (*P *> 0.05, Table 4).

**Table 4:** Comparison of the lipid and apolipoprotein levels in the genotypes of the 3'APOB-VNTR polymorphism between the Hei Yi Zhuang and Han populations

Nationality/Genotype	*n*	TC (mmol/L)	TG (mmol/L)	HDL-C (mmol/L)	LDL-C (mmol/L)	Apo A1 (g/L)	Apo B (g/L)
Hei Yi Zhuang							
VNTR-SS	186	4.39 ± 0.84	1.00 ± 0.47	2.22 ± 0.69	2.00 ± 0.56	1.41 ± 0.15	0.84 ± 0.20
VNTR-LS	80	4.56 ± 0.94	1.06 ± 0.49	2.38 ± 0.77	2.03 ± 0.42	1.42 ± 0.14	0.90 ± 0.24^#^
VNTR-LL	12	3.89 ± 0.70^†^	0.76 ± 0.16	1.83 ± 0.47^†^	2.09 ± 0.50	1.43 ± 0.08	0.69 ± 0.16^#††^
*F*	--	3.411	2.209	3.640	0.231	0.211	5.909
*P*	--	<0.05	>0.05	<0.05	>0.05	>0.05	<0.01

Han Nationality							
VNTR-SS	88	4.78 ± 0.88**	1.10 ± 0.62	2.50 ± 0.71**	2.00 ± 0.40	1.44 ± 0.11	0.92 ± 0.21**
VNTR-LS	74	4.57 ± 1.00	1.20 ± 0.54	2.40 ± 0.79	2.00 ± 0.42	1.42 ± 0.14	0.90 ± 0.24
VNTR-LL	10	4.68 ± 0.93*	1.12 ± 0.56*	2.39 ± 0.73*	2.01 ± 0.40	1.49 ± 0.05	0.91 ± 0.22*
*F*	--	1.011	0.599	0.396	0.003	1.654	0.160
*P*	--	>0.05	>0.05	>0.05	>0.05	>0.05	>0.05

TC, total cholesterol; TG, triglycerides; HDL-C, high-density lipoprotein cholesterol; LDL-C, low-density lipoprotein cholesterol; Apo A1, apolipoprotein A1; Apo B, apolipoprotein B; ^#^*P *< 0.05 in comparison withVNTR-SS genotype; ^†^*P *< 0.05 and ^††^*P *< 0.01 in comparison with VNTR-LS genotype; *P < 0.05 and **P < 0.01 in comparison with Hei Yi Zhuang

In order to evaluate the effects of allele frequency on the serum lipid levels, the three most common forms (HVE32, HVE34 and HVE36) in both ethnic groups were also be calculated separately. We found that the levels of TC in HVE34, and TG, LDL-C and Apo B in both HVE34 and HVE36 alleles were higher than those in HVE32 allele, whereas the levels of HDL-C in HVE32 allele were higher than those in both HVE34 and HVE36 alleles in Hei Yi Zhuang group. In Han populations, the levels of TC in HVE34, TG in HVE36, and LDL-C in both HVE34 and HVE36 alleles were higher than those in HVE32 allele (Table 5).

**Table 5:** Effects of HVE32, HVE34, and HVE36 alleles on the lipid and apolipoprotein levels between the Hei Yi Zhuang and Han populations

Nationality/Allele	*n*	TC (mmol/L)	TG (mmol/L)	HDL-C (mmol/L)	LDL-C (mmol/L)	Apo A1 (g/L)	Apo B (g/L)
Hei Yi Zhuang							
HVE32	214	3.98 ± 0.78	0.99 ± 0.48	2.47 ± 0.71	1.96 ± 0.55	1.43 ± 0.15	0.78 ± 0.20
HVE34	170	4.36 ± 0.91^##^	1.16 ± 0.52^##^	2.26 ± 0.68^##^	2.14 ± 0.64^##^	1.42 ± 0.14	0.91 ± 0.23^##^
HVE36	102	4.14 ± 0.73^†^	1.23 ± 0.46^##^	2.13 ± 0.59^##^	2.18 ± 0.57^##^	1.41 ± 0.11	0.86 ± 0.22^##^
*F*	--	10.221	10.241	9.955	0.231	0.751	17.741
*P*	--	<0.001	<0.001	<0.05	>0.05	>0.05	<0.001

Han Nationality							
HVE32	158	4.79 ± 1.02**	1.10 ± 0.74	2.52 ± 0.75	1.97 ± 0.65	1.43 ± 0.13	0.89 ± 0.20**
HVE34	182	4.54 ± 0.89^#^	1.23 ± 0.65	2.41 ± 0.80	2.32 ± 0.73^##^*	1.45 ± 0.15	0.94 ± 0.23
HVE36	102	4.72 ± 0.96**	1.36 ± 0.77^#^	2.35 ± 0.71*	2.24 ± 0.64^##^	1.44 ± 0.14	0.90 ± 0.21
*F*	--	3.078	4.220	1.714	11.716	0.854	2.471
*P*	--	<0.05	<0.05	>0.05	<0.001	>0.05	>0.05

TC, total cholesterol; TG, triglycerides; HDL-C, high-density lipoprotein cholesterol; LDL-C, low-density lipoprotein cholesterol; Apo A1, apolipoprotein A1; Apo B, apolipoprotein B; ^#^*P *< 0.05 and ^##^*P *< 0.01 in comparison with HVE32 allele; ^†^*P *< 0.05 in comparison with HVE34 allele; *P < 0.05 and **P < 0.01 in comparison with Hei Yi Zhuang

For further analysis of 3'APOB-VNTR allele frequency and its possible association with serum lipid levels, all 3'APOB-VNTR alleles were grouped into two main types (homozygotes and heterozygotes). The subjects of homozygote and heterozygote were 272 (49.6%) and 276 (50.4%) in Hei Yi Zhuang, and 324 (65.4%) and 172 (34.6%) in Han (*P *< 0.01); respectively. The levels of TC, TG, HDL-C and Apo B in Hei Yi Zhuang but not in Han were higher in homozygotes than in heterozygotes. There were no significant differences of Apo Al levels between the homozygotes and heterozygotes in both ethnic groups (*P *> 0.05, Table 6).

**Table 6:** Comparison of the lipid and apolipoprotein levels in the subjects of homozygote and heterozygote between the Hei Yi Zhuang and Han populations

Nationality	*n*	TC (mmol/L)	TG (mmol/L)	HDL-C (mmol/L)	LDL-C (mmol/L)	Apo A1 (g/L)	Apo B (g/L)
Hei Yi Zhuang							
Homozygote	272	4.62 ± 1.02	1.12 ± 0.71	2.37 ± 0.68	2.14 ± 0.55	1.43 ± 0.15	0.89 ± 0.19
Heterozygote	276	4.41 ± 0.87^††^	1.00 ± 0.46^†^	2.25 ± 0.71^†^	2.07 ± 0.52	1.42 ± 0.14	0.85 ± 0.21^†^
Han Nationality							
Homozygote	324	4.75 ± 1.01	1.18 ± 0.75	2.51 ± 0.73*	1.96 ± 0.45**	1.43 ± 0.14	0.95 ± 0.21**
Heterozygote	172	4.66 ± 1.00**	1.10 ± 0.62	2.46 ± 0.73**	2.02 ± 0.41	1.43 ± 0.13	0.91 ± 0.22^†^**

TC, total cholesterol; TG, triglycerides; HDL-C, high-density lipoprotein cholesterol; LDL-C, low-density lipoprotein cholesterol; Apo A1, apolipoprotein A1; Apo B, apolipoprotein B; ^†^*P *< 0.05 and ^††^*P *< 0.01 in comparison with homozygote of same nationality; *P < 0.05 and **P < 0.01 in comparison with Hei Yi Zhuang

### Relative factors of serum lipid levels

Multivariate logistic regression analysis showed that the levels of TC, HDL-C, Apo A1, and Apo B were positively correlated with age (*P *< 0.05–0.001), respectively. The levels of TG, HDL-C, and Apo A1 were positively correlated with alcohol consumption (*P *< 0.01–0.001), respectively, whereas the levels of LDL-C and Apo B were negatively associated with alcohol consumption (*P *< 0.001 and 0.05; respectively). The levels of HDL-C, LDL-C, and Apo A1 were positively correlated with sex (*P *< 0.05–0.001, Table 7), respectively. There is no significant correlation between the levels of TC, TG, HDL-C, LDL-C, Apo A1, and Apo B and cigarette smoking, BMI, pulse pressure (*P *> 0.05).

**Table 7:** Relationship between the lipid paramerers and relative factors

Lipid paramerers	Relative factors	Regression coefficient	Standard error	SRC	*t*	*p*
TC	Age (years)	0.011	0.004	0.230	2.764	0.006
TG	Alcohol consumption (g/day)	0.550	0.191	0.240	2.887	0.005
HDL-C	Age (years)	0.004	0.002	0.126	2.132	0.034
	Sex	0.172	0.072	0.161	2.387	0.018
	Alcohol consumption (g/day)	0.315	0.081	0.265	3.898	0.000
LDL-C	Sex	0.010	0.002	0.258	4.416	0.000
	Alcohol consumption (g/day)	-0.291	0.090	-0.188	-3.222	0.001
Apo A1	Age (years)	0.002	0.000	0.278	4.977	0.000
	Sex	0.051	0.019	0.175	2.745	0.006
	Alcohol consumption (g/day)	0.104	0.021	0.319	4.968	0.000
Apo B	Age (years)	0.003	0.001	0.310	0.310	0.000
	Alcohol consumption (g/day)	-0.057	0.026	-0.124	-2.140	0.033

TC, total cholesterol; TG, triglycerides; HDL-C, high-density lipoprotein cholesterol; LDL-C, low-density lipoprotein cholesterol; Apo A1, apolipoprotein A1; Apo B, apolipoprotein B; SRC, standardized regression coefficient

### Oligonucleotide sequence of the HVE 32 of the 3'APOB-VNTR gene

**Figure 4:**
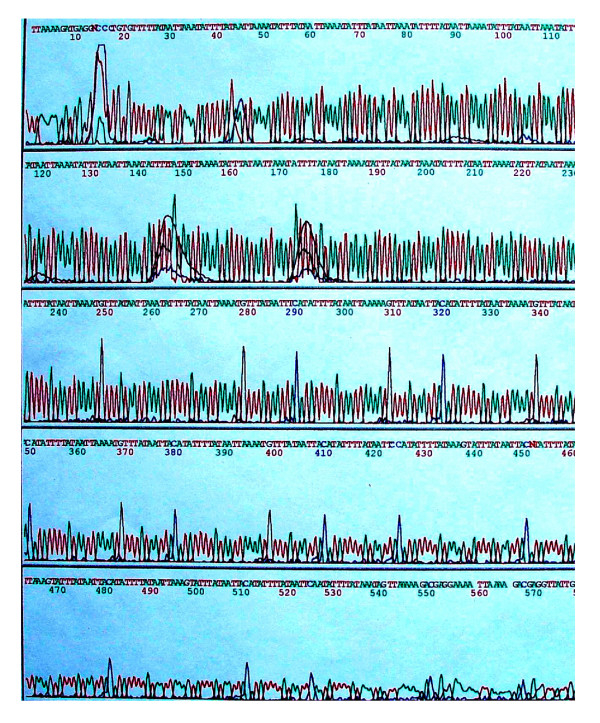
The oligonucleotide sequence of the HVE 32 of the 3'APOB-VNTR gene in the Hei Yi Zhuang.

The oligonucleotide sequence of the PCR product of HVE32 is shown in Table 8 and Figure 4, using the nomenclature initially proposed by Ludwig et al [39]. The first AT repeat sequence (ATAATTAAATATTTT) was defined as X type, and the second repeat sequence (ATAATTAAAATATTT) was defined as Y type. Thus, the oligonucleotide sequence of the HVE 32 of the 3'APOB-VNTR gene is XYY(XY)_5_(Xii Yii)_2_(Xii Yii)_4_Yii(Xi Yi)_2_Xii Xi.

**Table 8:** The oligonucleotide sequence of the HVE 32 of the 3'APOB-VNTR gene in the Hei Yi Zhuang

		5'-TTAAAAGATGAGGCCCTGTGTTTTT	
ATAATTAAATATTTT	ATAATTAAAATATTT	ATAATTAAAATATTT	ATAATTAAATATTTT
X	Y	Y	X
ATAATTAAAATATTT	ATAATTAAAATATTT	ATAATTAAAATATTT	ATAATTAAATATTTT
Y	X	Y	X
ATAATTAAAATATTT	ATAATTAAATATTTT	ATAATTAAAATATTT	ATAATTAAATATTTT
Y	X	Y	X
ATAATTAAAATGTTT	ATAATTAAATATTTT	ATAATTAAAATGTTT	ATAATTACATATTTT
Y	X	Yii	X
ATAATTAAAATGTTT	ATAATTACATATTTT	ATAATTAAAATGTTT	ATAATTACATATTTT
Yii	Xii	Yii	Xii
ATAATTAAAATGTTT	ATAAAGTATTT	ATAATTACATATTTT	ATAATTAAAGTATTT
Yii	Yii	Xii	Yi
ATAATTACATATTTT	ATAATTAAAGTATTT	ATAATTACATATTTT	ATAATTCAATATTTT
Xii	Yi	Xii	Xi
ATAAATAGTTAAAAAGACGAGGAAAATTAAAAGACGAGGTTATTGATCTCAGGAATTGTAT			
TCGCCCACGCGGAGAAGGA-3'			

Sequence: XYY(XY)_5_(Xii Yii)_2_(Xii Yii)_4_Yii(Xi Yi)_2_Xii Xi

## Discussion

The current study shows that the levels of serum TC, TG, LDL-C and Apo B in Hei Yi Zhuang were significantly lower than those in Han, whereas the levels of HDL-C in Hei Yi Zhuang were significantly higher than those in Han. There were no significant differences of Apo A1 levels between the two ethnic groups. These findings are in agreement with those of our previous studies [33,34]. It has been suggested that hyperlipidemia is a complex trait caused by multiple environmental and genetic factors. Genetic factors can account for approximately one-half of the variation in plasma LDL-C concentration in humans [43], considerable efforts have been made during the past decade to define mutations associated with hypercholesterolemia. Several studies have demonstrated that the variability of 3' Apo B polymorphism not only in distant populations but also, to a certain extent, in genetically relative ones [18-21]. Hei Yi Zhuang is a specific subgroup of Zhuang nationality in China. Strict intra-ethnic marriages have been performed from time immemorial in this nationality. Namely, only both man and woman are Hei Yi Zhuang can marry, and can not intermarry with the other subgroups of Zhuang or other nationalities [33,34]. Therefore, we are confident that the genetic factors are involved in the results of the present study. The hereditary characteristic and gene phenotypes of lipids in Hei Yi Zhuang may be different from those in Han.

In the present study, we showed that there were considerable differences of the 3'APOB-VNTR alleles between Hei Yi Zhuang and Han. Hei Yi Zhuang is devoid of the HVE56 and HVE58, whereas Han is short of the HVE48 and HVE62. The most frequent allele in Hei Yi Zhuang is VNTR32 (25.9%), followed by 34 and 30 repeats whose frequencies are 20.6% and 16.9% respectively, whereas the most frequent allele in Han is HVE34 (27.2%), followed by HVE32 (23.7%) and HVE36 (15.3%). The frequencies of HVE26, HVE30, HVE46, heterozygote, and short alleles were higher in Hei Yi Zhuang than in Han, whereas the frequencies of HVE34, HVE38, HVE40, homozygote, and long alleles were lower in Hei Yi Zhuang than in Han, suggesting that the allelic variation of the 3'APOB-VNTR polymorphism may have an association with various ethnic groups. Alavantic et al [22] found the HVE containing 34 repeats-HVE34 allele to be the most common one in both female and male samples from the Belgrade area, and that there was a lower frequency of the HVE>36 alleles. Verbenko et al [19] have reported that 25 alleles of the 3' ApoB minisatellite, ranging from 25 to 55 repeats, were detected in healthy unrelated individuals from the Russian Federation and the Republic of Belarus, in 10 populations from five ethnic groups: Russians, Byelorussians, Adygeis, Kalmyks and Yakuts. Heterozygosity indices were high and varied from 0.73 to 0.84. The distributions of alleles of this minisatellite in the Caucasoid populations (Russians, Byelorussians and Adygeis) had a bimodal character, whereas that for Mongoloid populations (Kalmyks and Yakuts) had a unimodal distribution. In a study of allelic frequency distribution at the hypervariable locus 3' to the Apo B gene in 5 human populations (Kacharis of northeast India, New Guinea Highlanders of Papua New Guinea, Dogrib Indians of Canada, Pehuenche Indians of Chile, and a relatively homogeneous Caucasian population of northern German extraction), Deka et al [44] also found 12 segregating alleles in 319 individuals. The two most frequent alleles, 37 and 39, were present in all the populations. In the present study, we found that alleles 56 and 58 were completely absent in Hei Yi Zhuang but present in Han, while alleles 48 and 62 were absent in Han but present in Hei Yi Zhuang. This clearly demonstrates the presence of allelic frequency variation in different populations. These association studies may be of some use when genetic factors are considered as one of the predisposing causes.

The potential relationships in humans between polymorphisms in the Apo B gene and the plasma or serum levels of Apo B-containing lipoproteins have been evaluated in a large number of studies [22-28]. Several reports documented associations between polymorphisms of the Apo B gene locus and LDL-C levels, but not one of these associations has been consistently observed in a large number of studies. Indeed, none of the common polymorphisms analyzed thus far has been generally accepted as a genetic marker of hyperlipidemia. Moreover, it is largely unknown whether any of the Apo B polymorphisms are linked directly to changes in Apo B metabolism. In the present study, we showed that the levels of TC, HDL-C and Apo B in Hei Yi Zhuang but not in Han were higher in VNTR-LS than VNTR-LL genotypes. The levels of TC in HVE34, and TG, LDL-C and Apo B in both HVE34 and HVE36 alleles were higher than those in HVE32 allele, whereas the levels of HDL-C in HVE32 allele were higher than those in both HVE34 and HVE36 alleles in Hei Yi Zhuang group. The levels of TC in HVE34, TG in HVE36, and LDL-C in both HVE34 and HVE36 alleles were higher than those in HVE32 allele in Han populations. The levels of TC, TG, HDL-C and Apo B in Hei Yi Zhuang were also higher in homozygotes than in heterozygotes. The reason for this discrepancy between the two ethnic groups is unclear. Our findings in Hei Yi Zhuang, however, are similar to those previously described in several studies [22-28]. In a healthy population sample from the Belgrade area, Alavantic et al [22] found that the HVE32 allele was associated with decreased serum levels of TC, LDL-C, and TG, and the 3'APOB-VNTR genotypes containing HVE34 and HVE36 alleles were associated with elevated serum levels of TC, LDL-C, and TG in males. In the obese people, Jemaa et al [23] found that the subjects with 50 repeat allele had significantly higher BMI, and subjects with 32 repeat allele had significantly higher HDL-C and ApoA1 levels. By the analysis of the healthy sample group, Garasto et al [24] found that the *S *(*Short*, <35 repeats) alleles lower the average values of serum TC and LDL-C while the alleles *M *(*Medium*, 35–39 repeats) and *L *(*Long*, >39 repeats) have no significant effect on the lipidemic phenotype. Studies that considered polymorphisms within the Apo B gene as risk factors for CHD have also reported conflicting results. Friedl et al [25] showed that the alleles containing 38, 44, 46, or 48 HVEs had an association with CHD. These alleles were also associated with elevated serum levels of TC and Apo B among patients and with elevated serum levels of TG among controls. Pan et al [26] showed CHD patients with two HVE36 alleles and no HVE32 alleles (the two most common forms) had significantly higher concentrations of LDL-C, Apo B, and TG, and significantly lower values of HDL-C and Apo A1 than the control group. The length of the Apo B 3' VNTR was not correlated with the plasma concentrations of any of the lipids. In a recent report, Yan et al [30] found that APOB gene 3'VNTR polymorphism exerts an impact on lipid metabolism and may contribute to the susceptibility to the development of CHD in Han Chinese. The frequency of 3'VNTR-B alleles (HVE = 38) in the CHD cases was higher than that of the controls. 3'VNTR-B allele was dependently related to TC levels. Compared with SS homozygotes, 3'VNTR-B allele carriers were associated with an increased risk of CHD. No significant differences in the internal structure and sequences of APOB gene 3'VNTR alleles were found between cases and controls. In a previous study, however, Hegele et al [45] found a significant correlation between Apo B gene polymorphisms and CHD, without any significant association with either LDL or VLDL. There was also no significant association between the 3'VNTR alleles and serum lipid levels in the Finnish population [31]. Similar results were reported in a porcine model of atherosclerosis in which an Apo B genetic variant was associated with atherosclerosis, despite normal lipid levels [46]. We surmise that the discrepancy may be related to the difference of ethnic groups.

In addition to the genetic factors, environmental factors such as diet may also paly an important role in determing the levels of these lipid phenotypes. In the present study, we showed that the levels of TC, HDL-C, Apo A1, and Apo B were positively correlated with age, respectively. The levels of TG, HDL-C, and Apo A1 were positively correlated with alcohol consumption, respectively, whereas the levels of LDL-C and Apo B were negatively associated with alcohol consumption; respectively. There is no significant correlation between the levels of TC, TG, HDL-C, LDL-C, Apo A1, and Apo B and cigarette smoking, BMI, and pulse pressure. Although Hei Yi Zhuang and Han live in the same region, the risk factors as mentioned above might be dissimilar. Great majority of Hei Yi Zhuang people live in the mountainous areas. The staple food is corn gruel or corn tortillas. On ordinary days, they are vegetarians. About 95% of the beverage is corn wine and rum. While Han takes rice as the staple food mostly. The standard of living in Han is higher than that in Hei Yi Zhuang. The intake of animal fat is more than that in Hei Yi Zhuang, and the body weight and BMI are also significantly higher than those in Hei Yi Zhuang [33,34]. For nearly 50 y it has been widely accepted that high-fat diets, particularly those that contain large quantities of saturated fatty acids, raise blood cholesterol concentrations and predispose individuals to cardiovascular disease [47]. In addition, the other major causation of different lipid levels between Hei Yi Zhuang and Han may relate to the excessive intake of corn. Corn contains abundant dietary fiber and plant high-quality protein [48]. Consumption of dietary fiber, specifically the soluble type, such a pectins and guar gum can result in a decrease of serum cholesterol levels in healthy and hyperlipidemic subjects [49]. Plant protein might raise the serum levels of HDL-C, and promote the transportation and excretion of free cholesterol. Corn oil is a kind of edible oils that is enriched with polyunsaturated fatty acid and monounsaturated fatty acid [50], and it is mostly used for cooking by Hei Yi Zhuang. A great deal of research has indicated that suitable intakes of polyunsaturated fatty acid and monounsaturated fatty acid can lower the serum levels of cholesterol and LDL-C [51,52]. A potential beneficial effect of dietary monounsaturated fatty acid on HDL-C has been suggested [52]. Corn oil could increase the ratio of HDL-C to TC and decrease the ratio of LDL-C to HDL-C [50].

In the present study, we also showed that the oligonucleotide sequence of the HVE32 of the 3'APOB-VNTR is somewhat different from that of a previous report [53]. In a previous structural and functional study of Apo B gene 3'VNTR alleles in Han Chinese, Chen et al [53] have described that the oligonucleotide sequence of the HVE32 of the 3'APOB-VNTR is XYX(XY)_5_(XYii)_2_(XiiYii)_3_XiiXiiYiii(XiiYi)_2_XiiXi or XYX(XY)_5_(XYii)_2_(XiiYii)_4_XiiXiiYiii(XiiYi)_2_XiiXi. In the present study, we showed that the oligonucleotide sequence of the HVE32 of the 3'APOB-VNTR in Hei Yi Zhuang is XYY(XY)_5_(XiiYii)_2_(XiiYii)_4_Yii(XiYi)_2_XiiXi, suggesting that the multiformity of the 3'APOB-VNTR polymorphism includes not only in the variable numbers of the repeat unit, but also in the variation in the oligonucleotide sequence within the repeat units as well as the variation in the arrangement of the repeat units [54].

## Conclusion

In conclusion, the current study shows that there were significant differences of the 3'APOB-VNTR polymorphism between Hei Yi Zhuang and Han. The levels of TC, HDL-C and Apo B in Hei Yi Zhuang but not in Han were higher in VNTR-LS than in VNTR-LL genotypes. The 3'APOB-VNTR genotypes containing HVE34 and HVE36 alleles were associated with elevated serum levels of TC, TG, LDL-C, and Apo B in Hei Yi Zhuang. The favorable lipid profiles of Hei Yi Zhuang might result from the effects of both environmental and genetic factors.

## Abbreviations

CHD, Coronary heart disease; Apo B, Apolipoprotein B; LDL, low-density lipoprotein; VLDL, very low-density lipoprotein; 3'APOB-VNTR, the variable number of tandem repeats region 3' of the Apo B gene; PCR, polymerase chain reaction; HVE, hypervariable elements; TC, total cholesterol; HDL-C, high-density lipoprotein cholesterol; TG, triglycerides; LDL-C, low-density lipoprotein cholesterol; VNTR, variable number of tandem repeats; BMI, body mass index; DNA, deoxyribonucleic acid; EDTA, ethylenediaminetetraacetate; SD, standard deviation; ANOVA, analysis of variance

## Competing interests

The author(s) declare that they have no competing interests.

## Authors' contributions

YR conceived the study, participated in the design, carried out the epidemiological survey, collected the samples, undertook genotyping, and drafted the manuscript; CG carried out the genotyping, performed the statistical analyses and helped to draft the manuscript; WY collaborated to the genotyping; LW and YD carried out the epidemiological survey, collected the samples, and helped to carry out the genotyping; PS participated in the design of the study and helped to carry out the genotyping. All authors read and approved the final manuscript.

## Pre-publication history

The pre-publication history for this paper can be accessed here:


